# Neonatal Imitation, Intersubjectivity, and Children With Atypical Development: Do Observations on Autism and Down Syndrome Change Our Understanding?

**DOI:** 10.3389/fpsyg.2021.701795

**Published:** 2021-08-27

**Authors:** Mikael Heimann, Emil Holmer

**Affiliations:** ^1^Infant and Child Lab, Department of Behavioural Sciences and Learning, Linköping University, Linköping, Sweden; ^2^Department of Behavioural Sciences and Learning, The Swedish Institute for Disability Research, Linköping University, Linköping, Sweden

**Keywords:** neonatal imitation, primary intersubjectivity, autism spectrum disorder, down syndrome, literature search

## Abstract

Almost all studies on neonatal imitation to date seem to have focused on typically developing children, and we thus lack information on the early imitative abilities of children who follow atypical developmental trajectories. From both practical and theoretical perspectives, these abilities might be relevant to study in children who develop a neuropsychiatric diagnosis later on or in infants who later show impaired ability to imitate. Theoretical in the sense that it will provide insight into the earliest signs of intersubjectivity—i.e., primary intersubjectivity—and how this knowledge might influence our understanding of children following atypical trajectories of development. Practical in the sense that it might lead to earlier detection of certain disabilities. In the present work, we screen the literature for empirical studies on neonatal imitation in children with an Autism spectrum disorder (ASD) or Down syndrome (DS) as well as present an observation of neonatal imitation in an infant that later was diagnosed with autism and a re-interpretation of previously published data on the phenomenon in a small group of infants with DS. Our findings suggest that the empirical observations to date are too few to draw any definite conclusions but that the existing data suggests that neonatal imitation can be observed both in children with ASD and in children with DS. Thus, neonatal imitation might not represent a useful predictor of a developmental deficit. Based on current theoretical perspectives advocating that neonatal imitation is a marker of primary intersubjectivity, we propose tentatively that an ability to engage in purposeful exchanges with another human being exists in these populations from birth.

## Introduction, Theory, and Motivation

Most published studies on imitation during the first months of life have focused on typically developing children. It might actually be *all* studies for imitation of facial gestures during the neonatal period. Researchers seem to avoid including children with any known risk factors for non-optimal development. It follows that reports often state that the children included in studies on neonatal imitation were born full-term and did not have any known medical complications. However, if imitation in the neonatal period is a real phenomenon affecting early social interaction and development, it becomes of uttermost importance to also investigate if children who show developmental deviances, either specifically in the social domain or more generally, differ from typically developing children in this ability. Here we present: (i) a comprehensive literature search focusing on published reports on imitation during the first year of life in children with an Autism Spectrum Disorder (ASD) or Down syndrome (DS); (ii) a home video observation that in our view might be interpreted as showing imitation during the neonatal period in a child later diagnosed with autism; and, (iii) a re-representation of previously published data on near-neonatal imitation in children with Down syndrome, observations that hitherto has gone largely unnoticed by the scientific community (Heimann et al., 1998).

The observation that infants might imitate already as newborns has been with us for a long time, and this ability has been reported by numerous studies since the 1970's (e.g., Meltzoff and Moore, 1977, 1983, 1989; Dunkeld, 1979; Maratos, 1982; Heimann and Schaller, 1985; Heimann et al., 1989; Kugiumutzakis, 1998; Nagy et al., 2013; for a recent meta-analysis, see Davis et al., 2021). Importantly, the existence of this phenomenon has been argued to reflect a rudimentary capacity for primary or innate intersubjectivity (i.e., Trevarthen, 1979, 2011a), that is, an ability to engage in intentional and purposeful exchanges with another human being (e.g., Trevarthen and Aitken, 2003; Rochat and Passos-Ferreira, 2009; Trevarthen, 2011a,b). Although we acknowledge the current debate around how to understand a newborn child's imitative-like responses (e.g., Oostenbroek et al., 2016; Jones, 2017), this paper rests on the assumption that those responses are best described as an act of neonatal imitation (e.g., Simpson et al., 2014; Meltzoff et al., 2018; Heimann and Tjus, 2019). In line with this, we also assume that this early act of imitation reflects a potential expression of primary intersubjectivity and that we need a better understanding of its place in development for children following both typical and atypical developmental pathways. Our hypothesis that newborn imitation is an example of an early social motive that signifies an intersubjective capacity is furthermore anchored in the works by several different theoreticians over the years (e.g., Bråten, 1998; Reddy, 2008; Rochat and Passos-Ferreira, 2009), but foremost on Trevarthen's groundbreaking ideas as exemplified in these two quotes:

“Most remarkably, before a baby has competence for handling and exploring non-living objects, he or she shows sensitive awareness of the motive states and feelings of other persons who offer to interact in well-timed contingency with what the infants expresses, and the baby reacts in intricately adaptive interpersonal ways to human expressions, often imitating, but not just by imitating.” (Trevarthen and Aitken, 2003, p. 112).

“Infant human beings imitate other humans, not just to act like them, but to enter into a communicative and cooperative relationship with them by some transfer of the feeling of body action.They can, in this way, start building understandings that may serve later to identify a particular companion in the meaning of a shared world” (Trevarthen, 2011a, p. 124).

If neonatal imitation is one of the first signs of innate or primary intersubjectivity as proposed by Trevarthen (1979, 2011b), Bråten (1998), Reddy (2008), and Kugiumutzakis and Trevarthen (2015), then the question also arises to what degree children following an atypical developmental trajectory would show an early imitative ability. In other words: Is this capacity of the neonate associated primarily with typical development, or is it an ability that also can be observed among infants, following an atypical developmental trajectory?

**Figure 1:**
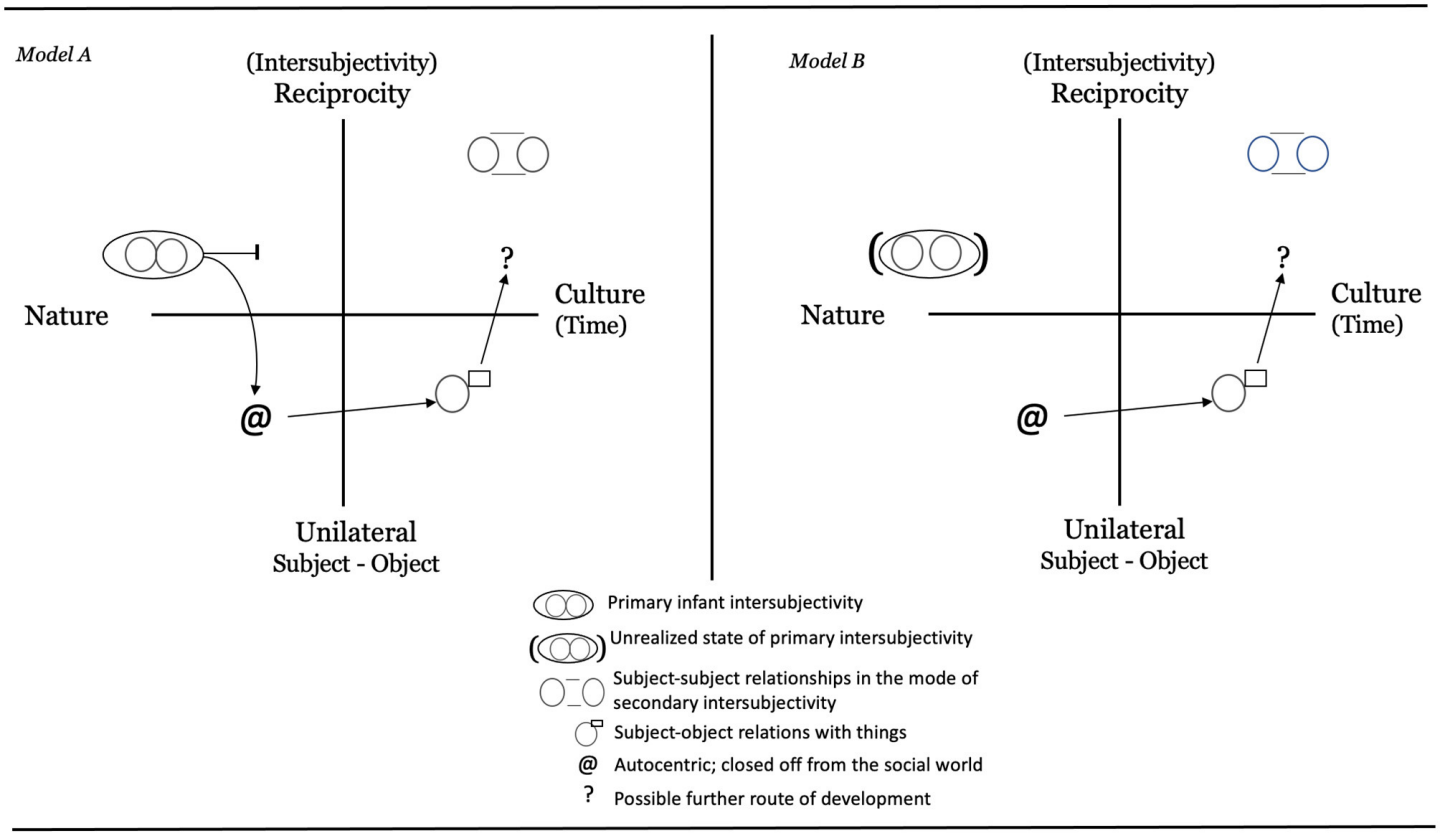
Two theoretical models describing the infants state of mind with respect to intersubjectivity at birth for a child later diagnosed with autism. *Explanations: Model A* describes an initially typical development, the infant is born with a capacity of primary intersubjectivity, autism emerges later. *Model B* describes a starting state that is different from the beginning. A child within the autism spectrum does not have the same capacity to intersubjectivity at birth, the mind is not dialogical from the start (Adopted from Heimann, 1998, p. 100–101. Reprinted with permission).

For autism, imitation has often been highlighted as one of the capacities that develops slowly and possibly represents a core deficit (Sigman et al., 2004; Volkmar et al., 2005; Nadel, 2006, 2014; Rogers, 2006; Vanvuchelen et al., 2011; Gowen, 2012; Vivanti and Hamilton, 2014). Imitation has been viewed as important for children with ASD because it “supports a sense of interpersonal connectedness and mutuality” (Sigman et al., 2004; p. 224), capacities that people with autism often find difficult. This aspect plus the fact that imitation is an important tool for learning through observation have made imitation training an important part of various training and intervention programs for children with autism (see Schreibman et al., 2015; Spjut Jansson et al., 2020). As one central example, Rogers and Pennington (1991) included imitation as one of the early deficits in their theory on autism. According to them, neonatal imitation is an early social competence that would be missing in newborn children that later develop autism. In a subsequent theoretical attempt, Heimann (1998) outlined two possible developmental routes for children with autism. Building on Bråten's (1988, 1998) theoretical formulations that, from the beginning, the mind is both dialogical and intersubjective, two hypothetical models of development were formulated (see Figure 1).

Heimann (1998) reasoned that neonatal imitation, as a marker of primary intersubjectivity, may or may not be observable in infants who later show signs of autism or other developmental disabilities. In one scenario, neonatal imitation is linked to more advanced imitation and intersubjective abilities at later stages in development, which leads to the expectation that neonatal imitation should be impaired in infants with ASD. Thus, a diminished imitation ability in a neonate might be a sign of an unfulfilled capacity to partake in early intersubjective social encounters. A second scenario noted by Heimann (1998) is that neonatal imitation might not have a direct relationship to later imitative ability and intersubjective development. In this scenario, neonatal imitation might be observable in infants with autism or other developmental disorders that are associated with impairments in the social domain. This could mean that mechanisms for primary intersubjectivity are not impaired from birth in these populations and, instead, that atypicality in relating to another person emerges later as other layers of intersubjectivity develop.

One problem in determining whether a lack of the ability for neonatal imitation is an early marker of autism is that the condition is not usually diagnosed before the child is several years old and rarely before 2 years of age (Ozonoff et al., 2015; Goldstein and Ozonoff, 2018; Zwaigenbaum et al., 2020). Even if many parents report retrospectively that they did note problems during the first year of life, it has not been possible to pinpoint an exact cluster of behaviors that makes it possible to reach a definite diagnosis early in life (but see Wetherby et al., 2021 for an early identification protocol). An additional problem is that some children with autism show a typical developmental trajectory from birth to about 12 or 18 months of age whereafter they start to lose abilities (Ozonoff et al., 2008). An example of such a regressive pattern might be a child who, after being able to point and utter his or her first words, suddenly stops both pointing and talking.

Davis and Crompton (2021) highlight the growing insight that the social difficulties associated with autism or other neurodiverse conditions “are at least in part bidirectional” (p. 652). They also argue for researchers to use a difference perspective, in contrast to the more traditional deficit model. This leads to the need for a research framework that charters socio-cognitive abilities in detail and that avoids preconceived expectations of what to expect or not to expect from autistic people or other neurodiverse groups. Within the scope of this paper, this means that we must acquire a better knowledge of the competencies of, for example, infants with the risk of developing autism. If we ever will be able to understand how the different social and communicative abilities of autistic persons evolve, we must differentiate between problems residing within the individual from problems arising from “a mismatch of interpersonal dynamics” (Davis and Crompton, 2021; p 650), as proposed by the dialectical mismatch hypotheses (Bolis et al., 2017).

For children with DS, early development differs from most children with autism (although the two syndromes can also overlap). DS is a chromosomal aberration usually diagnosed at birth or shortly thereafter and almost all children with this syndrome end up having a mild to moderate intellectual disability (Udwin and Dennis, 1995; Di Nuovo and Buono, 2011; Ostermaier, 2019). Since the diagnosis is made early, one would have expected that some studies on imitation at birth or shortly thereafter in this group would have been conducted. But this seems not to be the case. One of the few comprehensive and longitudinal studies of the early psychological development focusing on children with DS is the study by Dunst (1990) that describes sensorimotor development over the first 3 years of life.

The mean age of the nine children constituting the youngest group in Dunst's sample was 2.9 months, and they displayed an almost typical level of imitation according to the Uzgiris-Hunt scale (Uzgiris and Hunt, 1975). However, imitation of facial gestures like tongue protrusion or mouth opening used in studies of imitation in newborn children were not included. Dunst used Piaget's theory when chartering the early development of children with DS and concluded that this group follows a similar developmental trajectory as typical children, although at a slower rate. The development of the youngest group, children younger than 4 months, was almost on par with typical infants (Dunst reported a developmental quotient of 85), but they were clearly below average when they reached their first birthday (DQ = 62). This “slowing down phenomena was most pronounced for vocal imitation” according to Dunst (1990; p. 224).

While studies on neonatal imitation in humans to date have almost exclusively focused on healthy infants (e.g., Meltzoff et al., 2018; Davis et al., 2021), our goal is to provide observations relevant for children developing along atypical trajectories that might affect how the capacity for primary intersubjectivity develops. We will do this through three different paths presented as three different studies:

1. A search through scientific databases for papers on imitation in infants at-risk for ASD and/or DS during the first year of life with a primary focus on the neonatal period or early infancy up to 3 months of age;
2. Presenting tentative observations from a brief home video on imitation-like responses in a neonate developing along an autism trajectory; and
3. In a re-use of published findings, we present a more in-depth analysis of how five one-month-old children with DS respond in an imitation experiment.

## Study 1. Literature Search

The psycINFO, PubMed, and Scopus databases were searched for publications in English on the topic of imitation in populations with DS or ASD younger than 2 years of age. Searches of articles were conducted by the second author (E.H.) on December 3, 2020, in psycINFO and PubMed, and on February 26, 2021, in Scopus (for search terms and limiters, see Supplementary Materials). We did not set any limit for publication date.

**Figure 2:**
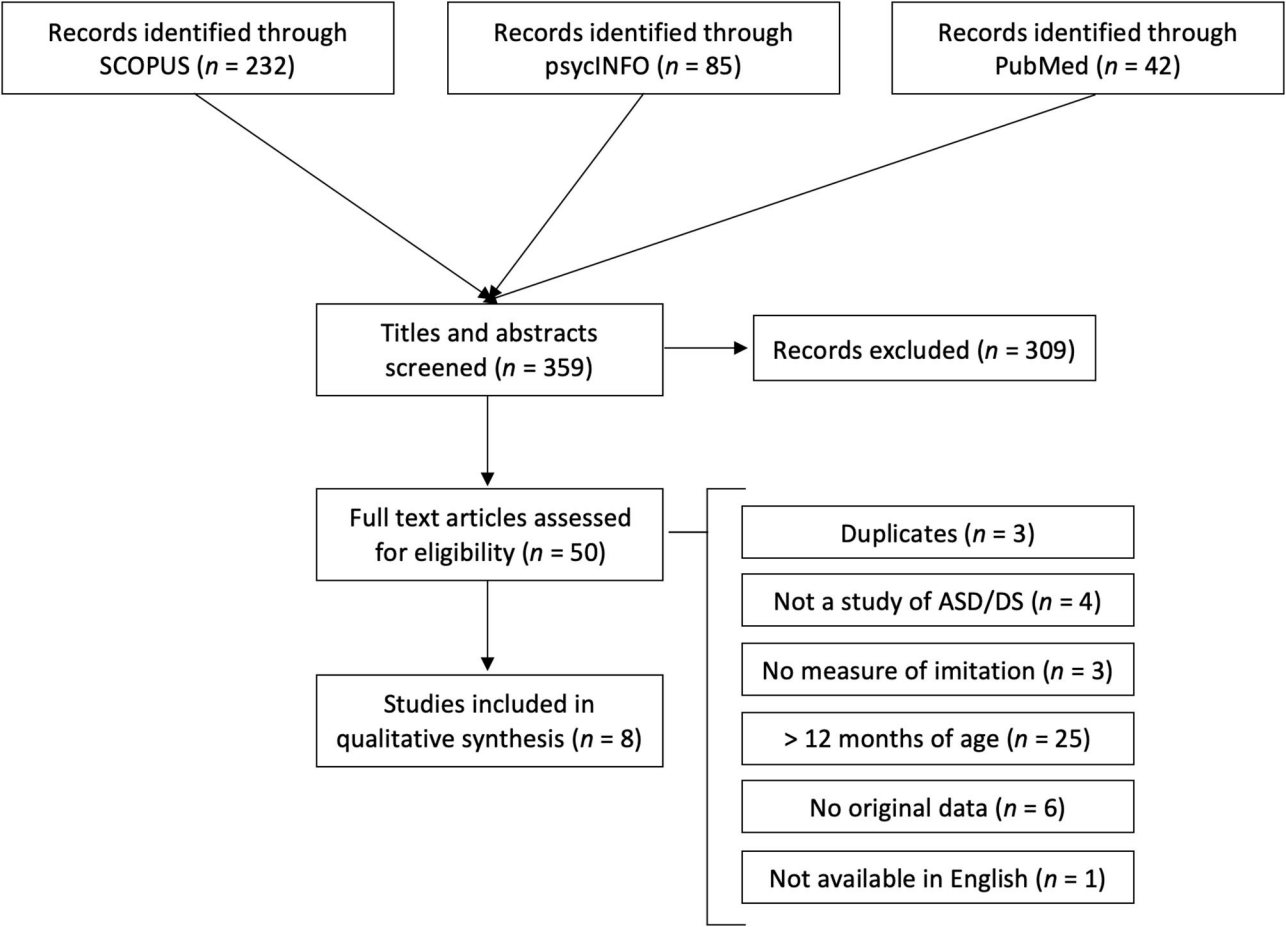
Flow diagram of the literature search.

A flow diagram of the search including the four phases recommended by Liberati et al. (2009) is presented in Figure 2. The search resulted in 85 records in psycINFO, 42 in PubMed, and 232 in Scopus. After review of titles and abstracts, 50 records were kept for full-text review. The most common reason for exclusion was that the publication was not a study of imitation (e.g., mimicry of medical conditions), studied a non-human population (e.g., rodents), or included participants that were older than the targeted populations. Full-text records were reviewed for inclusion independently by both authors. Inclusion criteria were that the publication described an original empirical study with at least one imitation task (e.g., experimental procedure, observational methods, behavioral ratings), and included participants with DS or ASD that were younger than 12 months of age.

After full-text reviews, four articles were deemed eligible for inclusion by both authors, and we agreed to reassess an additional five articles due to uncertainties in study design. Four of these five articles were included after reassessment, and both authors agreed on excluding the fifth. The reasons for exclusion are listed in Figure 2. The eight articles kept for this review are described in Table 1.

**Table 1:** Description of identified articles in the literature search.

**Study**	***N***	**Participant description**	**Imitation assessment**	**Imitation results**
**Autism spectrum disorder**				
Bolton et al. (2012)	86	Children diagnosed with autism spectrum disorder at 11 years of age. Participants were part of a large sample (*N* = 14,541) followed from the age of 6 months.	Parental questionnaire at 6 and 11 months.	Responses to questions referring to imitation/play at age 6 months was not a significant predictor of diagnosis at age 11 years. Potentially a predictor of later degree of autistic trait (but overlap between instruments).
Dawson et al. (2000)	1	A boy diagnosed with autism spectrum disorder at 1 year of age (assessed on the Autism Diagnostic Interview, Lord et al., 1994). First notes/observations at 2.5 months of age.	Notes from medical journal at 9 months.	Notes suggesting that the child does not play imitation games with parents.
Keemink et al. (2021)	18	Infants (11 boys) with at least one older sibling who had an autism spectrum disorder diagnosis. Six participants were 6 months old and 12 were 9 months old.	Participants and controls performed a gaze-contingency task, looking at faces that turned from a neutral expression to an emotional. Spontaneous imitation in response to emotional facial expressions was coded.	Participants were less likely to imitate facial emotion expressions than the control group.
Neimy et al. (2020)	3	Infants (one boy) aged 7, 8, and 11 months, with at least one older sibling who had an autism spectrum disorder.	During a baseline before the introduction of an intervention, vocal imitation (“echoics”) was coded based on video observation of interactions between the mother and the child.	One child showed evidence of vocal imitation during baseline.
Receveur et al. (2005)	18	Children (13 boys) diagnosed with autism spectrum disorder after the age of 4 years, and split into two groups based on high (*n* = 8)/low (*n =* 10) developmental quotient (as indicated by a score above/below 50 on the Brunet-Lezine Scales, Brunet and Lezine, 1983). Observations started at 10–12 months.	Participants were filmed by their parents during the two first year of life. Imitation was scored by using the Imitation Disorder Evaluation scale (Malvy et al., 1999) on video observations.	At 10–12 months, there was no difference in imitation score between children with high and low developmental quotient.
Sanefuji and Yamamoto (2014)	21	Children (16 boys) with scores above cut-off for autism spectrum disorder on ADOS (Lord et al., 1989) at 24 months. Participants were part of a larger sample of 54 high-risk children (i.e., younger siblings of children with an autism spectrum disorder). First observation at 11 months.	Imitation ability was assessed on experimental tasks, including object manipulation (Meltzoff, 1988), gesture imitation (Smith and Bryson, 2007), and movement imitation (Bekkering et al., 2000; Rogers et al., 2003).	At 11 months of age, no statistically significant differences were observed between participants above the cut-off on ADOS at 24 months compared to those below.
**Down syndrome**				
Heimann et al. (1998)	8	Infants (7 boys) diagnosed with Down syndrome. Successful testing performed at 1 (*n =* 5), 3 (*n =* 7), and 4 (*n =* 7) months of age.	Tongue protrusion and mouth opening were presented to the infant by an experimenter and responses were video recorded. Imitation was coded from videos, defined as a matching response that exceeded the observed rate of non-matching responses.	Evidence of imitation of tongue protrusion but not mouth opening was reported at 1 month of age. At 3 and 4 months of age, no evidence of imitation was observed at the group level, although some individuals imitated either tongue protrusion or mouth opening.
Bauer and Jones (2015)	3	Infants (2 boys) with Down syndrome, aged 7, 8, and 9 months.	During a baseline before the introduction of an intervention, an experimenter produced vocal utterances that the infant was prompted to imitate (e.g., “Say, ‘mmm'.”). Imitation was assessed from video recordings, was coded based on video observation of interactions between the mother and the child.	Two infants showed limited evidence of vocal imitation during baseline.

Studies included children with DS (*n* = 2), ASD (*n* = 4), or children from a high-risk population for ASD (i.e., younger sibling to a child with an ASD, *n* = 2). The earliest measure of imitation that was reported was from an age of 1 month, and the same participants were also observed at 3 and 4 months of age (Heimann et al., 1998). Age spans in the rest of the studies were in the range of 6–11 months. The largest study included 86 children with a diagnosis, and the smallest was a single-case study. Two studies reported results from an intervention, with baseline measures of spontaneous imitation in mother-child interaction, while the other studies used parental questionnaire (*n* = 1), notes from medical records (*n* = 1), or experimental procedures (*n* = 3). Four of the studies had a control or comparison group, and one of these (Keemink et al., 2021) reported that 6–9-month-old-infants at high-risk for ASD were less likely than a control group to spontaneously imitate facial emotion expressions; in the three other studies, no between-group differences were detected under the age of 12 months.

## Study 2. Imitation In A 3-Day Old Child Later Diagnosed as Autistic

The following text tells the story of Marcus, a boy with ASD. When he was 3-days old, his parents used a smartphone to take a video of him as parents often do, and Marcus' mother later provided this home video to the first author (M.H.). The very brief home video shows the father modeling tongue protrusion and how Marcus responds. Marcus received an autism diagnosis before his third birthday, and his story is briefly described in this section.

### Birth and Early Development

Marcus was born at term (gestational age: 38 weeks). His birthweight was within the expected range (3,030 g) and there were no signs of asphyxia or other immediate complications. In fact, his Apgar scores were perfect (10, 10, 10). However, the pregnancy had not been uncomplicated. His mother had spent some periods in the hospital due to infections and pneumonia. In spite of his perfect Apgar scores, Marcus was diagnosed with serious complications a couple of hours after birth: early onset GBS sepsis (Group B streptococcal septicemia), a condition that can seriously influence a child's health and further development (Libster et al., 2012).

After treatment and a prolonged stay in the hospital before the parents were allowed to take him home, Marcus seemed to develop as expected during his first year of life. The parents were acquainted with what to expect from a child during the first year of life (Marcus was their third child), and they did not note any atypical signs early on. He made adequate eye contact according to his mother, he developed pointing as expected, and he uttered his first word before his first birthday. However, the situation changed shortly after his first birthday. It became more and more difficult to maintain eye contact with him, and his interest in other people decreased sharply. Parallel to this, he became less verbal, and eventually he stopped talking. Instead, he became more focused on objects, puzzles, and YouTube video clips. These behavioral changes began to worry his parents and, when he was 19 months old, his mother found a screening instrument online, the Modified Checklist for Autism in Toddlers (MCHAT; Robins et al., 2001). She answered the items in the checklist and received a score of 21 out of 23 with the following summary and recommendation: “This score suggests that your toddler is at elevated risk for autism or another developmental disorder and should be evaluated by a specialist for early intervention services.”

### First Clinical Evaluation and Diagnosis

The family contacted the health services who referred them to a neuropsychiatric clinic that initiated an evaluation shortly before Marcus' second birthday. The team was made up by a child psychiatrist, neuropsychologist, speech and hearing therapist, and a special education teacher. Some excerpts from the neuropsychological and medical examination provides a good context for understanding the grounds for his diagnosis:

The psychologist notes that Marcus speaks no words and does not use gestures but is able to clearly express both joy and when he dislikes something. He is easily frustrated, but it is relatively easy to get him back on track. He does not initiate any interaction and does not respond to any invitation. He uses his mother's hand when needing any help. The psychologist also notes that he gives eye contact only once during the whole assessment.

The psychologist used mainly two instruments during this initial assessment: Vineland Adaptive Behavior Scales II (Sparrow et al., 2005), a parental questionnaire, and a developmental test, the Griffiths Scale of Child Development I (Alin-Åkerman and Nordberg, 1983). The results from Griffiths can be translated into age equivalents (AE) indicating the age level that corresponds to the responses a child gives. Marcus is 24 months old when evaluated, and the result is an uneven profile. His scores are close to his biological age within three of the areas included in the Griffiths test: the gross motor, eye-hand coordination, and performance scales (AE's 17–22 months). In contrast, he shows a clearly protracted development on the two scales sensitive to language, communication, and social development (AE's 6 $\frac{1}{2}$ and 11 $\frac{1}{2}$ months). The result from the Vineland parental interview showed that Marcus' adaptive abilities were affected. His most severe problem area was his communicative abilities whereas his motor abilities were judged to be at age level.

The result from the psychological evaluation is confirmed both by the detailed analysis of his language and communicative development conducted by the speech and language therapist and by evaluation with the Autism Diagnostic Observation Schedule (ADOS; Lord et al., 1989, 2000) performed by the special education teacher. The Children's Global Assessment Scale (C-GAS) (Shaffer et al., 1983; Lundh et al., 2010) was added by the child psychiatrist. Marcus received a score of 38 which indicates “major impairment in functioning in several areas and unable to function in one of these areas” (e.g., at home, in preschool or with peers; Shaffer et al., 1983, p. 1229). In conclusion, the child psychiatrist sees the same pattern as his team members and concludes that Marcus fulfills the criteria for classic autism and intellectual disability according to DSM-IV-TR (American Psychiatric Association, 2000) and ICD-10 (World Health Organization, 1990). Specifically, the child psychiatric evaluation established that Marcus fulfilled all DSM-IV-TR criteria for qualitative impairment in social interaction, two out of four criteria for qualitative impairments in communication, and three out of the four criteria listed for repetitive and stereotyped patterns of behavior (American Psychiatric Association, 2000, see Table 2).

**Table 2:** The criteria for an autism diagnosis according to DSM-IV-TR that Marcus fulfilled according to the clinical evaluation.

	**Abridged DSM-IV criteria for autism**	**Marcus' evaluation**	
1.	*Qualitative impairment in social interaction^*a*^*		
	a. impairment in the use of non-verbal behaviors (e.g., eye-to-eye gaze)	Yes	
	b. failure to develop peer relationships	Yes	
	c. lack of sharing enjoyments with other people	Yes	
	d. lack of social/emotional reciprocity	Yes	
2.	*Qualitative impairments in communication^*b*^*		
	a. delay or lack of development of spoken language	Yes	
	b. inability to initiate conversations		No
	c. idiosyncratic use of language		No
	d. lack of pretend play or imitative play	Yes	
3.	*Restricted patterns of behavior, interests, and activities^*b*^*		
	a. preoccupation with restricted pattern of interest	Yes	
	b. inflexible adherence to routines	Yes	
	c. stereotyped motor mannerisms (e.g., hand flapping)	Yes	
	d. preoccupation with parts of objects		No

a *Two criteria must be met*;

b *At least one criterion must be met*.

### Home Video Suggesting a Capacity to Imitate

**Figure 3:**
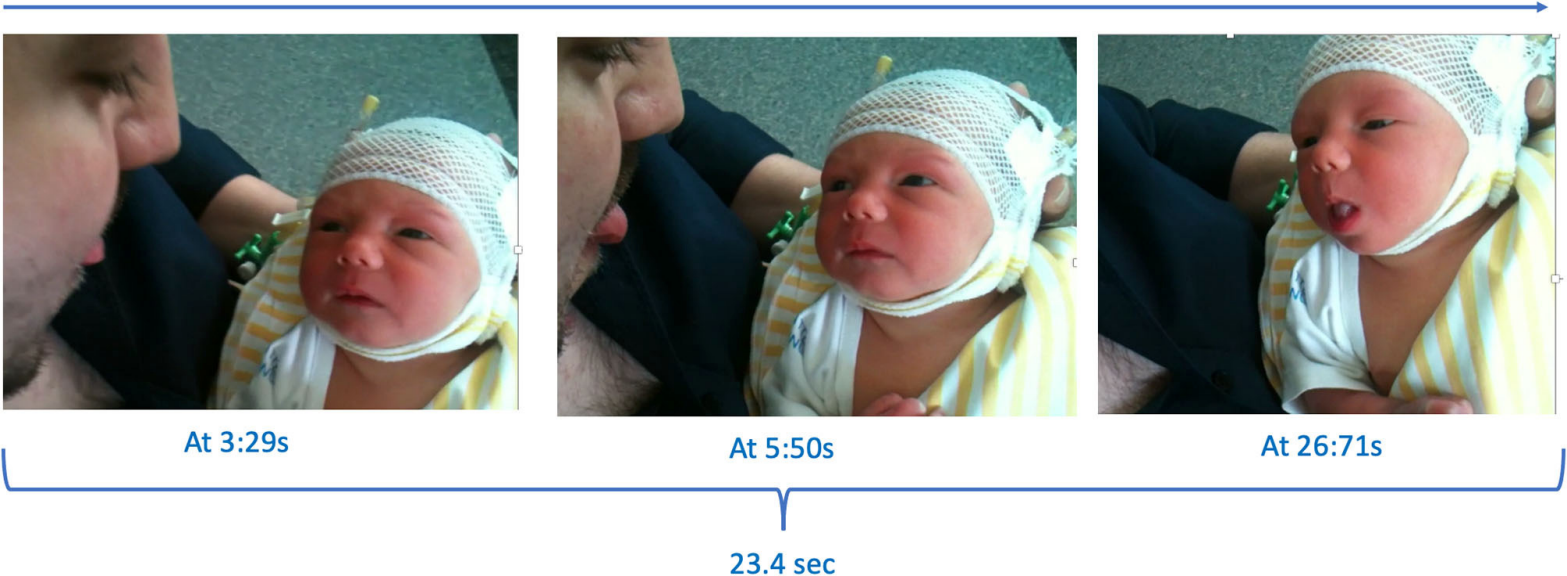
A sequence of still photos (courtesy of the family) from the home video showing Marcus' response. Tongue protrusion is defined as a clear forward movement of the tongue even if the tongue was not protruded beyond the outer part of the lips (see Heimann and Tjus, 2019).

When Marcus was 3 days old, his mother used her smartphone to take photos and some brief videos of him together with his father. One of these videos show the father sticking out his tongue when holding Marcus, who seems both calm and attentive although slow in his movements. The segment is only about 30 s long (see Figure 3 and Table 3), during which his father presents six tongue protrusions and Marcus responds with three. The criteria for judging tongue protrusion in this case follows earlier publications by accepting as a minimum criterion that a clear forward movement of the tongue is noted although the tongue might not be protruded beyond the back edge of the lower lip (e.g., Meltzoff and Moore, 1983; Oostenbroek et al., 2016; Heimann and Tjus, 2019). Marcus responses were coded by the first author (M.H.) and independently by two other researchers. No statistical analysis was possible since there was no control gesture presented to Marcus and no section of the video that could be used as a possible baseline measure.

**Table 3:** A sequential overview of the home video showing Marcus responding with tongue protrusion (TP) to his fathers' modeling.

**Timeline^**a**^ (s)**	**Father (model)**	**Marcus (response)^**b**^**
0	Looks at Marcus	Looks slightly away
3	Presents first TP	Looks at F
6	Second TP	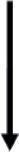
8	Third TP	
10	Looks at Marcus	Eyes closed
12	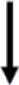	Looks at F
13	Fourth TP	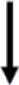
15	Looks at Marcus	Looks away
18	Fifth TP	Looks at F
22	Sixth TP	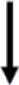
23	Looks at Marcus	TP
25	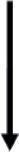	TP
26		TP
27	Smiles	Looks away

a *Time averaged to whole seconds*;

b *TP defined as a clear forward movement of the tongue even if the tongue was not protruded beyond the outer part of the lips*.

### Discussion

We acknowledge that this is anecdotal evidence that must be interpreted with much caution (see Ozonoff et al., 2011, for a comparison between home videos and clinical evaluations), but it is notable from the video that Marcus shows no other facial movements, such as mouth opening, during this brief episode. His first response is a tongue protrusion that comes more than 20 s after his father protruded his tongue for the first time. Regardless if we define Marcus' response as imitation or not, the video does show that a 3-day old infant later diagnosed with autism is able to match tongue protrusion. And he does so in a way that, in many aspects, mimics how neonatal imitation of facial gestures has been studied and described in published studies (e.g., Heimann and Tjus, 2019). If neonatal imitation is linked with later imitation and social responsiveness, this observation suggests that it is probably not a direct link–at least not so for children with autism since imitation is a skill that often is part of the initial training used in comprehensive preschool behavioral training programs for children with autism (e.g., Vismara and Rogers, 2010; Ingersoll and Meyer, 2011; Spjut Jansson et al., 2020). Furthermore, if we take the observation as a true sign of neonatal imitation, then it suggests that a child with autism (maybe all children with autism), who we know have a different and problematic relationship with the social world as they develop, might be no less social as newborns than “typically” developing children.

## Study 3. Imitation In One-Month Old Infants With Down Syndrome

### Background

There seems to be a dearth of studies on the socio-cognitive abilities of children with DS at birth, as evident from the literature search described above. We could not identify a single study investigating imitation among infants with DS during their first 2–3 weeks of life. The only study that came close was conducted by one of the authors (M. H.) more than 20 years ago (Heimann et al., 1998) with the goal to study facial imitation over the first 3 months in an attempt to parallel previous published observations on typical infants.

The initial plan, when the study was conceived in the 1990's, had been to carry out the first observation when the children were still within the neonatal period, that is, before 1 month of age. However, due to both medical and psychological reasons, this became impossible. Children with DS often require extra medical support and/or evaluations directly after birth. For the parents, even if they might have known beforehand that their expecting child had DS, the early neonatal period usually becomes a time of adjustment when focus is on other issues than research. In the end, we succeeded to recruit a group of eight children with DS (see Heimann et al., 1998) born between gestational weeks 36–39 (*Mdn* = 39) and, for five of them, we were able to conduct the first observation close to the neonatal period when they on average were 37 days old (*SD* = 11.0; range 25–52). The focus here will be only on the five children (all male) observed around 1 month of age.

### Method

All observations took place in the home of the children using light-weight portable video equipment. The parents were often present in the room during the observation and the sessions did not begin until the child was judged to be awake and alert. TP or MO were presented to the child by an experimenter and all gestures were presented during a pre-set interval of 20 s followed by a response time of equal length. This sequence was repeated three times, giving each child a total observation time of 120 s (*M* = 122.1 s; *SD* = 9.4). The order of presentation was randomized, and the experimenter did not know beforehand which gesture to start with. By definition a TP occurred whenever a clear forward movement was detected, even if the tongue only passed the posterior part of the lip (similar to the definition used for Marcus in the previous section). MO “was defined as a clear and visible separation of the lips that was judged to meet the criteria of a definite change. Some children kept their mouths open over extended periods of time which was not accepted as a MO. A clear change had to take place” (Heimann et al., 1998; p. 781). Furthermore, no concurrent forward trust of the tongue was accepted nor was yawning. All videos were coded blindly by two research assistants and the obtained Kappa coefficient was 0.92. Imitation was defined behaviorally: an individual child was judged to imitate if the frequency of matching responses exceeded the observed number of non-matching responses.

### Results

We conducted three separate analyses: (1) the overall result across the complete 2-min period; (2) the result for the three modeling periods; and (3) the result for the three response periods. Based on the current knowledge at the time when the study was conducted (in the 1990's) that children with DS develop “in the same sequence as that followed by normal children” (Hodapp and Zigler, 1990; p. 10), we hypothesized that we would find that our DS group displayed imitation similar to what had been observed for typical children. Statistically we used two-tailed tests (Wilcoxon and sign test).

**Figure 4:**
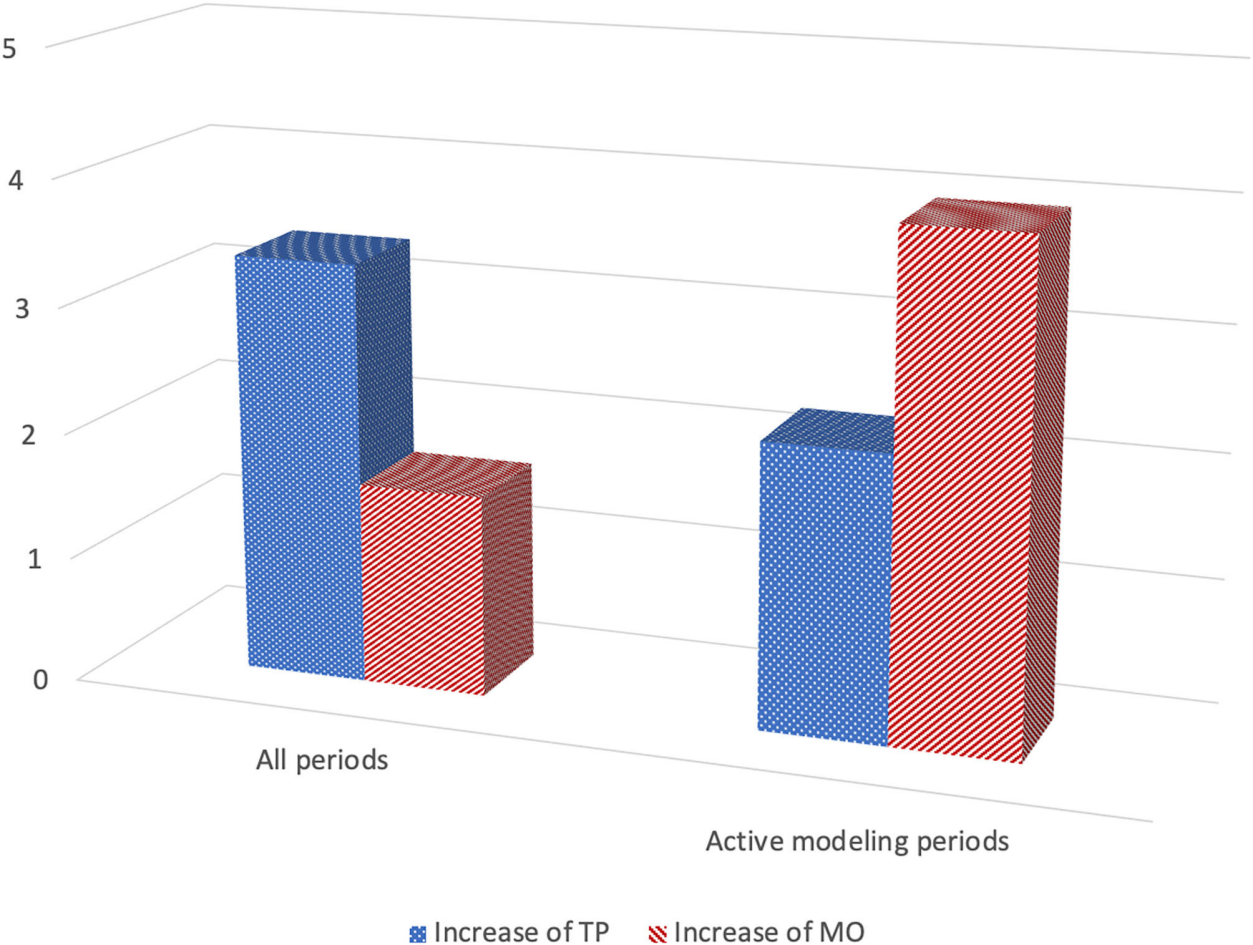
Imitation in infants with Down syndrome (*N* = 5) at 1 months of age: Mean rate increase of tongue protrusion (TP) and mouth opening (MO) after modeling of each gesture during all periods of the experiment (6 × 20 s) as well as only during the active modeling periods (3 × 20 s). See text for more details (based on Heimann et al., 1998, Tables 3, 4).

We found support for imitative-like responses when the whole period and the modeling periods were analyzed but not when focusing only on the response periods (Heimann et al., 1998). The most convincing indication of imitation was found when only the modeling periods were analyzed (see Figure 4). The frequency of TP increased on average with 2.3 responses (range 1.2–3.8) when TP was modeled in comparison with the observed frequency of TP when modeling mouth opening (Sign test; *z* = 2.23; *p* = 0.025, Wilcoxon; *z* = 2.02; *p* < 0.05). The pattern for MO was similar, the frequency of MO increased with on average 4.0 mouth openings (range 1.6–7.4) when MO was modeled in comparison with the number of MOs observed after modeling of TP (Sign test; *z* = 2.00; *p* = 0.046; Wilcoxon; *z* = −1.75, *p* = 0.08).

Individually, all five children imitated TP during modeling. The pattern for MO was slightly different, four of the children imitated, while the fifth child displayed a pattern of no change, the frequency of MO stayed the same in both conditions. Putting it differently, none of the children responded with what could be described as a contra-imitative pattern, for instance displaying the highest frequency of MO when TP is modeled. At least not when only the modeling periods were analyzed.

### Discussion

Even if this small study indicates that infants with DS seems able to display near-neonatal imitation under some conditions it is worth noting that the children responded a bit different to what we had previously observed for typical infants (Heimann et al., 1989; Heimann and Tjus, 2019). Their mean rate of responses, especially so for TP, often exceeded what we have previously observed for infants during the neonatal period (Heimann et al., 1998). Furthermore, the different result for the modeling and response periods tentatively suggests that children with DS are helped by having stimuli in sight in order to respond. When the modeling stops and the response period start, they lose focus and fail to differentiate their response.

It is not possible to generalize or draw any definite conclusions from a study of only five infants. Despite this and the fact that children with DS might be an even more heterogenous group than typical infants, the findings from the 1-month-old observation are relatively straightforward. During the periods when the gestures were actively modeled all five children imitated TP and four out of five MO. No child displayed a strong non-imitative pattern of increasing the frequency of the control gesture (e.g., TP) in comparison with the gesture being modeled (e.g., MO). The only child not imitating showed no change, he opened his mouth an equal number of times both when MO and TP were modeled. The paper on which this summary is based did “conclude that children with Down syndrome show an early capacity for imitation similar to that usually expected for normal infants during the first few weeks of life” (Heimann et al., 1998; p. 783). Today we would also cautiously propose that children with DS show signs of a dialogical mind (Bråten, 1988) and a capacity for primary intersubjectivity (Trevarthen, 2011a) already at 1 months of age. We do however not know if this ability is there already at birth or not.

## General Discussion

The aim of this endeavor has been to explore what is known from empirical studies on the existence of imitation or imitation-like responses near birth or during the first year of life among children with non-typical trajectories. We have done this through three different venues: by searching published reports via three different databases; by presenting a previously unpublished observation on facial imitation in a 3-day-old infant that later received an ASD diagnosis; and finally by a renewed presentation of previously published observations on imitation in five 1-month-old children with DS. Based on the observations reported here, we tentatively propose that the little empirical evidence that exist implies that children with ASD and DS have a similar capacity for neonatal imitation as do typically developing children and, thus, an innate capacity for primary intersubjectivity. However, our most critical suggestion is that there is a great need of studies investigating neonatal imitation in atypical populations.

Searching Scopus, PubMed, and PsycInfo for papers on neonatal imitation in atypical populations resulted in 50 papers receiving a full-text review, of which 42 were excluded in the end for not fulfilling our criteria (e.g., focused on diseases, non-human populations, or included participants older than 12 months). Of the eight articles included, only two focused on DS, and the only article that described development from birth in a case of a boy with ASD did not comment on imitation before the age of 9 months (Dawson et al., 2000). The one article describing imitation around the first month of life was the one by the first author on this paper (M.H.), described in detail above. Thus, our literature search shows that almost no empirical research on neonatal imitation exists in the target populations of this paper. This is particularly surprising in the case of ASD, since imitation is assumed to be impaired in this population and a possible precursor of later deviant social development (Rogers and Pennington, 1991; Vanvuchelen et al., 2011). The only documented observation that we have been able to identify is the case of Marcus described in this paper and based on that, we do not currently see any support for the hypothesis that imitation is absent in the neonatal period for children on the track to develop ASD. Clearly, more research is highly needed to test this assumption.

**Figure 5:**
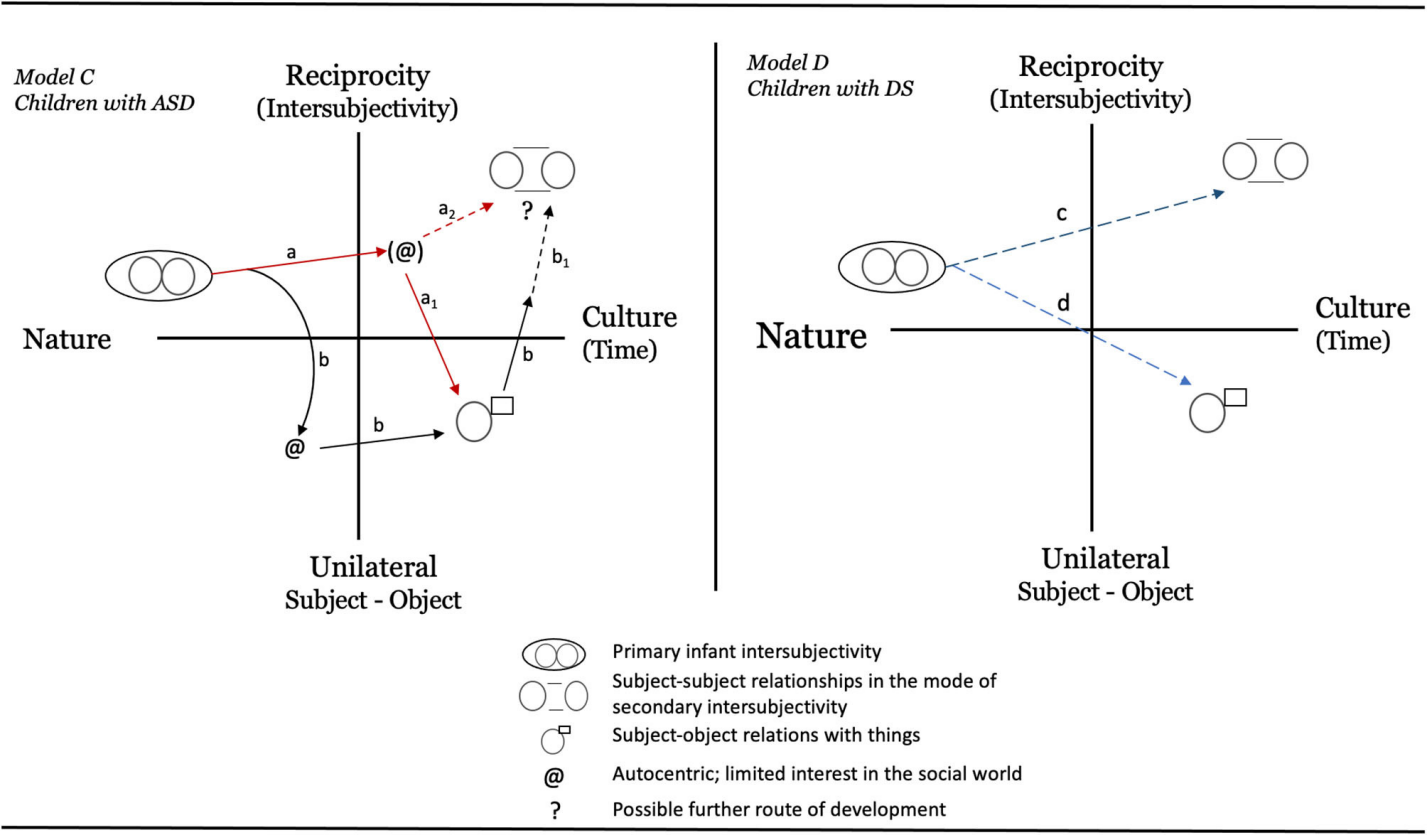
Proposed new models based on the observations presented in the text. Model C represents ASD and Model D describes tentative paths for DS. *Explanations* of paths: *Model C:* (1) Path *a* depicts a child with autism developing along a typical trajectory over the first 12–18 months where after a halt in development takes place and the child loses social abilities previously mastered (indicated by path *a*_1_). This child might eventually find a renewed social interest (maybe via an intervention) and move via path *a*_2_ to develop a capacity for subject-subject relationships. (2) Path *b* depicts a child lagging behind in social ability already during the first year but note that even this child has the capacity for primary intersubjectivity at birth. The child might stay in a mindset that is occupied with subject-object relations but may also later move toward the social world (illustrated by *b*_1_). *Model D:* Paths *c* and *d* are almost identical to the paths in the original model presented by Bråten (1996) for typically developing children. As in Model C, the infant has the ability to act in a complementary way and the participants step into each other's dialogic circle already from the start. The figure also illustrates different developmental paths for relations with people as compared to objects. The main difference from Bråten's original model is that instead of solid lines, the lines here are dashed in an attempt to illustrate that children with DS usually show a slower pace of development and might not reach the expected end state.

For ASD, the home video of Marcus imitating TP when only 3-days-old raises questions about the starting state of a child developing along an autistic path. True, it is only anecdotal evidence based on a very brief video. But even as such, the observation challenges our knowledge of how ASD develops over time. One might argue that Marcus is unique and that the observation says very little about children with ASD overall. Still, to our knowledge this is the first documented observation of its kind. It has, for instance, direct bearing on the two models based on Bråten's theory (Bråten, 1988, 1998) that Heimann (1998) outlined. Based on the video of Marcus, we suggest that Model B should be dismissed in favor of Model A, which, by allowing for an initial state of primary intersubjectivity, probably is closer to the truth. However, even this model is limited since it does not take the heterogeneity of autism into account (see Fountain et al., 2012; Georgiades et al., 2013; Mottron and Bzdok, 2020). We therefore suggest an updated model, Model C, as illustrated in Figure 5. This new model outlines two possible trajectories for children later receiving an ASD diagnosis. Path *a* illustrates a child developing typically over the first one to one and a half years, whereafter a regressive pattern occurs meaning that some social or communicative skills are lost (Parr et al., 2011; Thompson et al., 2019). The other trajectory, path *b*, shows an early deviance from typical development, notable well before the child's first birthday. Note that none of the paths deviate at birth. This is not to say that genetic and biological factors might not be different from typical children early on–although imaging studies so far have been unsuccessful in identifying biological indicators of autism in infants below 6 months of age (see Shen and Piven, 2017). Instead, Model C suggests that any differences in social and communicative skills between children with autism and non-autistic children will not be easily detectable this early on a behavioral level. This proposal is in line with what we saw in the literature search, in which few studies reported a difference between children at risk for ASD and typically developing children during the first year of life.

Berger (1990, p. 137) concludes that most “infants with DS are able to enter into reciprocal interactions with their parents soon after birth” even if some delay can be detected for early behaviors such as smiling, vocalizing, and eye contact. In Berger's sample, mutual eye contact displayed a delay of 2.5 weeks in onset. This is also reflected in Model D (Figure 4) that illustrates two main trajectories for how subject-subject (path *c)* and subject-object (path *d)* relationships might develop for children with DS. Note that these paths are similar to what we would expect for typical children (Dunst, 1990; Bråten, 1996; Heimann, 1998). The main difference being that children with DS usually show a much slower pace of development and might not reach the expected end state.

According to the findings presented here and in the Heimann et al. (1998) paper, it would, in our view, be wise to add imitation to the list of early social behaviors that children with DS might display. A capacity which signals that most children with DS have the capacity to establish relationships with the quality of primary intersubjectivity at birth or very shortly after. All children in the DS sample discussed here had at least a month of experience before they took part in the experiment. Thus, we cannot disentangle an innate capacity from rapid learning during their first month of life.

From a theoretical point of view, the observations provided suggest the possibility that both ASD and DS children are born with a mind that has an ability for primary intersubjectivity that makes it possible for them to enter into early dialogues as described by, among others, Reddy (2008). In this way, their starting state seems similar to what we expect to observe in typical neonates. This further implies that the difficulties we see later in development most probably are not caused by a lack of ability for rudimentary social interaction but, instead, emerge when the conditions for interaction changes, possibly when typical children start to engage in secondary intersubjectivity. Another possibility is that for children with ASD, the non-social world at some stage becomes more “attractive” than the social. As suggested by Davis and Crompton (2021), the evolving difficulties will, for some autistic children, also be influenced by non-optimal bidirectional processes that repeatedly create a mismatch between interacting partners, in this case within the early parent-infant dialogues. This is not to say that autism is caused by caregivers' responses, only that also children on a path to autism is affected by continuous positive or negative social experiences.

There are some limitations to consider when interpreting the findings reported here. To start, the empirical base for drawing any conclusion varies between our three studies. The literature search rests on a comprehensive scan across three central and relevant databases that allow us to be more definite about the scarcity of studies investigating imitation in newborn and young children with ASD or DS. The empirical support for the existence of an actual capacity to imitate for children with ASD or DS is however much weaker, close to non-existent. For ASD, we have a single home video that is <1 min long and only the parents' reassurance that the situation was spontaneously filmed. According to the information the parents provided they had never tried to elicit imitation before the video was recorded. Furthermore, a further limitation is that the video only contains documentation of imitation of TP and no sequence when the father was passive that could have been used as comparison. However, the fact that no other responses than TP is produced by Marcus during the brief video adds to the quality of the observation.

For DS, we have taken a new look at already published observations on imitation. Although the data consists of a small number of children (*n* = 5), they represent all published observations on near neonatal imitation for this group to date, as shown by our literature review. It should however be noted that the DS infants responded differently to what we usually observe for typical infants. The participating DS infants produced a higher frequency of TP in comparison with data from studies on typical infants (Heimann et al., 1998). We do not know if this is a difference that is significant when it comes to the capacity to imitate, but it should remind us that the abilities of newborn children with DS might not be identical to typical infants.

Regarding the literature search, although we worked systematically we might have overseen relevant search terms or additional databases. Perhaps even more critical, we did not sweep the field for gray literature (e.g., unpublished dissertations, null findings in file drawers), and we might therefore have failed to include relevant literature. There is reason to believe that a publication bias might exist in the area of neonatal imitation since there is skepticism in the field whether the phenomenon exists (e.g., Oostenbroek et al., 2016). This, in combination with the general issues in conducting studies on atypical populations (e.g., small populations, large heterogeneity, medical complications), might lead to an unwillingness to include clinical groups like ASD or DS in studies on neonatal imitation. Consequently, this leads to an absence of such studies in published literature which, in our view, is very unfortunate since it hinders theoretical advancements. A recent meta-analysis did conclude that there is evidence to suggest that neonatal imitation exists (Davis et al., 2021), and therefore we believe that it is warranted to focus more on individuals from atypical populations as we continue to investigate it.

## Conclusion

Our study highlights the lack of empirical support for the notion that neonates with ASD or DS do not have a capacity to imitate. Although resting on limited evidence, we believe that our observations instead tentatively point in the direction of an imitative capacity also for children that follows a developmental trajectory different from neurotypical children. Thus, all newborn children are probably ready for social encounters, and during their first interactions with another human being, they will use their capacity for primary intersubjectivity to establish reciprocity.

It is also striking that our literature search revealed so few studies on neonatal or early imitation in children with ASD or DS. For ASD, this is despite the fact that imitation in general and neonatal imitation specifically have been theoretically in focus for a long time. The lack of studies including infants or neonates with DS is also surprising since this is a group of children identified very early, often before birth. Thus, it ought to be possible for clinicians to gather larger samples systematically over time. This would give us a more solid ground from which to evaluate how children with DS are capable of imitation early in life. For ASD it is more difficult to study neonatal imitation directly, but one possibility could be to include imitation in the neonatal period in future studies on siblings to children with ASD.

## Data Availability Statement

The datasets presented in this article are not readily available because the Down syndrome data has been published earlier and the home video is not public material. However, the procedure for the literature review is available in a Supplementary Materials. Any requests regarding the Down syndrome data or the home video should be addressed to the corresponding author.

## Ethics Statement

The studies involving human participants were reviewed and approved by The Ethical Review Board, Göteborg University for the Down Syndrome children. For the ASD home video, the parents gave consent and approved the final text. This study was conducted according to the World Medical Association's ethical principles as outlined in the Helsinki declaration. Written informed consent to participate in this study was provided by the participants' legal guardian/next of kin, including consent for the publication of any potentially identifiable images or data included in this article.

## Author Contributions

The study was conceived by MH. Data were collected and analyzed by MH and EH. The manuscript was written jointly by both authors.

## Conflict of Interest

The authors declare that the research was conducted in the absence of any commercial or financial relationships that could be construed as a potential conflict of interest.

## Publisher's Note

All claims expressed in this article are solely those of the authors and do not necessarily represent those of their affiliated organizations, or those of the publisher, the editors and the reviewers. Any product that may be evaluated in this article, or claim that may be made by its manufacturer, is not guaranteed or endorsed by the publisher.
