# Supplementary material for: Neonatal Imitation, Intersubjectivity, and Children With Atypical Development: Do Observations on Autism and Down Syndrome Change Our Understanding?
Source: Front Psychol. 2021 Aug 27;12:701795. doi: 10.3389/fpsyg.2021.701795 (PMC8430258; doi:10.3389/fpsyg.2021.701795)
Supplement: Supplementary file 1 [file Data_Sheet_1.docx]

**Supplementary material: Search terms**

**SCOPUS search terms**

*Search performed February 26, 2021*

ABS ( imitat* OR mimic* OR emulat* OR "behaviour matching" OR "behavior matching" OR "gesture matching" OR "behaviour copying" OR "behavior copying" OR "gesture copying " ) AND ABS ( autism OR asd OR "autism spectrum disorder" OR "asperger syndrome" OR "high functioning autism" OR "down syndrome" OR "trisomy 21" OR "down's syndrome" OR down's OR trisomy OR downs OR "downs syndrome" ) AND ABS ( neonat* OR newborn OR infant OR baby  ) AND ( LIMIT-TO ( LANGUAGE , "English" ) )

Results: 232 records

**psycINFO search terms**

*Search performed December 3, 2020*

AB ( imitat* OR mimic* OR emulat* OR behaviour matching OR behavior matching OR gesture matching OR behaviour copying OR behavior copying OR gesture copying ) AND AB ( autism OR asd OR autism spectrum disorder OR asperger syndrome OR high functioning autism OR down syndrome OR trisomy 21 OR down's syndrome OR down's OR trisomy OR downs OR downs syndrome ) AND AB ( neonat* or newborn or infant or baby )

*Added limiters* Language: English, Population group: Human

Results: 85 records

**PubMed search terms**

*Search performed December 3, 2020*

("imitat*"[Title/Abstract] OR "mimic*"[Title/Abstract] OR "emulat*"[Title/Abstract] OR "behaviour matching"[Title/Abstract] OR "behavior matching"[Title/Abstract] OR (("gestural"[All Fields] OR "gesturally"[All Fields] OR "gesture s"[All Fields] OR "gestured"[All Fields] OR "gesturer"[All Fields] OR "gesturers"[All Fields] OR "gestures"[MeSH Terms] OR "gestures"[All Fields] OR "gesture"[All Fields] OR "gesturing"[All Fields]) AND "matching"[Title/Abstract]) OR (("behavior"[MeSH Terms] OR "behavior"[All Fields] OR "behavioral"[All Fields] OR "behavioural"[All Fields] OR "behavior s"[All Fields] OR "behaviorally"[All Fields] OR "behaviour"[All Fields] OR "behaviourally"[All Fields] OR "behaviours"[All Fields] OR "behaviors"[All Fields] OR "pattern"[All Fields] OR "pattern s"[All Fields] OR "patternability"[All Fields] OR "patternable"[All Fields] OR "patterned"[All Fields] OR "patterning"[All Fields] OR "patternings"[All Fields] OR "patterns"[All Fields]) AND "copying"[Title/Abstract]) OR "behavior copying"[Title/Abstract] OR (("gestural"[All Fields] OR "gesturally"[All Fields] OR "gesture s"[All Fields] OR "gestured"[All Fields] OR "gesturer"[All Fields] OR "gesturers"[All Fields] OR "gestures"[MeSH Terms] OR "gestures"[All Fields] OR "gesture"[All Fields] OR "gesturing"[All Fields]) AND "copying"[Title/Abstract])) AND ("autism"[Title/Abstract] OR "asd"[Title/Abstract] OR "autism spectrum disorder"[Title/Abstract] OR "asperger syndrome"[Title/Abstract] OR "high functioning autism"[Title/Abstract] OR "down syndrome"[Title/Abstract] OR "trisomy 21"[Title/Abstract] OR "down s syndrome"[Title/Abstract] OR "down's"[Title/Abstract] OR "trisomy"[Title/Abstract] OR "downs"[Title/Abstract] OR "downs syndrome"[Title/Abstract]) AND ("neonat*"[Title/Abstract] OR "newborn"[Title/Abstract] OR "infant"[Title/Abstract] OR "baby"[Title/Abstract])

*Added limiters* Language: English, Population group: Human

Results: 42 records
